# Feasibility of exercising adults with asthma: a randomized pilot study

**DOI:** 10.1186/1710-1492-8-13

**Published:** 2012-08-03

**Authors:** Amy Boyd, Celeste T Yang, Kim Estell, Craig Tuggle MS, Lynn B Gerald, Mark Dransfield, Marcas Bamman, James Bonner, T Prescott Atkinson, Lisa M Schwiebert

**Affiliations:** 1 Department of Pediatrics, University of Alabama, 1918 University Boulevard, Birmingham, AL, 35294-0005, USA; 2 Department of Biostatistics, University of Alabama, 1918 University Boulevard, Birmingham, AL, 35294-0005, USA; 3 Department of Cell, Developmental, and Integrative Biology, University of Alabama, 1918 University Boulevard, Birmingham, AL, 35294-0005, USA; 4 The Mel and Enid Zuckerman College of Public Health, University of Arizona, Tucson, AZ, USA; 5 UAB Lung Health Center, University of Alabama, 1918 University Boulevard, Birmingham, AL, 35294-0005, USA; 6 Department of Medicine, University of Alabama, 1918 University Boulevard, Birmingham, AL, 35294-0005, USA

## Abstract

**Background:**

Aerobic exercise appears to have clinical benefits for many asthmatics, yet a complete understanding of the mechanisms underlying these benefits has not been elucidated at this time.

**Purpose:**

The objective of this study was to determine feasibility for a larger, future study that will define the effect of aerobic exercise on cellular, molecular, and functional measures in adults with mild-moderate asthma.

**Design:**

Recruited subjects were randomized into usual care (sedentary) or usual care with moderate intensity aerobic exercise treatment groups.

**Setting / Participants:**

Nineteen adults with mild-moderate asthma but without a recent history of exercise were recruited at the UAB Lung Health Center, Birmingham, AL.

**Intervention:**

The exercise group underwent a 12 week walking program exercising at 60 – 75% of maximum heart rate (HR_max_). Subjects self-monitored HR_max_ levels using heart rate monitors; exercise diaries and recreation center sign-in logs were also used.

**Main outcome measures:**

Functional measures, including lung function and asthma control scores, were evaluated for all subjects at pre- and post-study time-points; fitness measures were also assessed for subjects in the exercise group. Peripheral blood and nasal lavage fluid were collected from all subjects at pre- and post-study visits in order to evaluate cellular and molecular measures, including cell differentials and eosinophilic cationic protein (ECP).

**Results:**

Sixteen subjects completed the prescribed protocol. Results show that subjects randomized to the exercise group adhered well (80%) to the exercise prescription and exhibited a trend toward improved fitness levels upon study completion. Both groups exhibited improvements in ACQ scores. No changes were observed in lung function (FEV1, FEV1/FVC), cell differentials, or ECP between groups.

**Conclusions:**

Results indicate that a moderate intensity aerobic exercise training program may improve asthma control and fitness levels without causing asthma deterioration in adult asthmatics. As such, these findings demonstrate the feasibility of the study protocol in preparation for a larger, clinical trial that will elucidate the functional consequences of aerobic exercise on asthmatic cellular and molecular responses.

## Background

Asthma is characterized by the symptoms of wheezing, chest tightness, dyspnea and cough, and by the presence of reversible airway narrowing and/or airway hyperresponsiveness (AHR) to bronchoconstrictor stimuli. Although multifactorial in origin, asthma is considered an inflammatory process that is the result of an inappropriate immune response to common stimuli. Previous studies have demonstrated that increased levels of inflammatory mediators, such as serum eosinophilic cationic protein (ECP), correlate positively with asthma exacerbations and worsening [1,2].

Increasing evidence indicates that decreased physical activity may play a role in the severity and increasing prevalence of asthma [3]. We and others have reported that, in murine asthma models, repeated bouts of aerobic exercise at a moderate intensity attenuate both airway inflammation and hyperreactivity significantly [4-7]. Furthermore, several clinical studies suggest that aerobic exercise training, as a part of a pulmonary rehabilitation program, improves asthma control and overall physical fitness of asthmatics and reduces their disease-related hospital admissions [3,8-10]. In accordance with these studies, the American College of Sports Medicine (ACSM) and the American Thoracic Society (ATS) recommend the implementation of low to moderate intensity aerobic exercise for asthmatic patients [11,12]. Specifically, the ACSM suggests walking or other forms of exercise that utilize large muscle groups 3–5 days per week at 50% of maximal exertion. The ATS recommends exercising at 60 to 75% of maximal work rate for 20 to 30 minutes per day 2 to 5 days per week, and our study follows the ATS guidelines for exercise.

Despite these reports and recommendations, however, the physiologic basis for the clinical improvement that many asthmatics experience with a regular exercise program is not understood fully. The objective of this pilot study was to determine feasibility for a larger, future study that will define the effect of moderate intensity aerobic exercise on cellular, molecular, and functional measures in adults with mild-moderate severity asthma. Nineteen subjects were randomized into two treatment groups: usual care (sedentary) or usual care with moderate intensity aerobic exercise. Subjects in the exercise group underwent a 12 week walking program exercising at 60 – 75% of maximum heart rate (HR_max_). Outcome indicators included functional (lung function, ACQ, fitness), cellular (circulating cell differentials), and molecular (pro-inflammatory mediators, including ECP) measures. Results show that subjects randomized to the exercise group adhered well (80%) to the exercise prescription and exhibited a trend toward improved fitness levels as compared with sedentary controls. Both groups exhibited improvements in Asthma Control Questionnaire (ACQ) scores. No changes were observed in lung function (FEV1, FEV1/FVC), cell differentials, or pro-inflammatory mediator levels, including ECP, between groups. Despite these observations, we maintain that this current study demonstrates the feasibility of the protocol in preparation for a larger clinical trial that will elucidate the functional consequences of aerobic exercise on cellular and molecular responses in asthmatic patients.

## Methods

### Subjects

**Figure 1:**
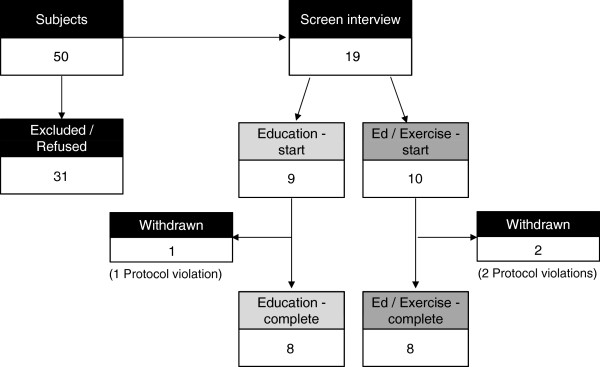
**CONSORT figure of subject screening and recruitment.** Fifty subjects were interviewed; thirty-one were excluded or refused. The remaining subjects were randomized into education and education with exercise treatment groups. A total of three subjects (1 – education; 2 – education/exercise) were withdrawn from the study because of protocol violations.

This randomized, parallel group proof of concept study was approved and monitored by the UAB Institutional Review Board. Subjects were recruited from the University of Alabama at Birmingham (UAB) Lung Health Center’s Asthma Clinical Research Database from March 2009 through June 2011. Adults with mild-moderate persistent asthma (as defined by the NAEPP guidelines [13]) with at least a 12% FEV_1_ reversibility were eligible for enrollment. A physician diagnosis of asthma and documentation of reversible airflow obstruction was utilized to exclude patients with other causes of dyspnea. Individuals who underwent aerobic exercise regularly (3 or more times per week for more than 20 minutes at a time) during any of the six months prior to the study were not eligible for enrollment in order to facilitate the examination of exercise-mediated effects on asthmatic responses. In addition, individuals who smoked within 6 months from the start of the exercise protocol or with greater than a 10 pack year smoking history were excluded in order to exclude patients with chronic obstructive pulmonary disease (COPD). Individuals with other major illnesses, including coronary artery disease, congestive heart failure, stroke, severe hypertension, immunodeficiency states, or other conditions that would have interfered with participation in the study or with the proposed measurements were not eligible. In order to facilitate high adherence and data collection rates, individuals who were unable or unwilling to provide consent, perform the exercise protocol, provide pre- and post-study measurements, be contacted via telephone, or who intended to move out of the area within 6 months from the start of the study were excluded. Figure 1 illustrates the number of subjects screened and enrolled in the study.

### Interventions

Subjects were recruited by the study coordinator and randomly assigned to either usual care or usual care with moderate intensity aerobic exercise treatment groups (Figure 1). Permuted variable size block randomization developed by the biostatistician was used to allocate subjects to the two study arms. The variable block size prevented exact knowledge of the next randomization assignment while maintaining equal allocation of subjects to the study arms throughout the study. All subjects were provided with two zippered pillow encasements and one mattress protector (Royal Heritage). These items were membrane free materials with a pore size less than 5% to reduce subject exposure to dust mite and dander in their bedding. In addition, study subjects randomized to the moderate intensity aerobic exercise group received a 3 month free membership to a local exercise facility at the time of the initial visit. This allowed the study subject a secure and safe environment in which to perform the exercise protocol. This strategy was designed to reduce adverse events associated with allergen exposure as well as prevent drop outs due to difficulty obtaining a location within which to perform the exercise protocol.

#### Usual care asthma education

All subjects received a brief (approximately 30 minute) coordinator-led educational intervention at the UAB Lung Health Center. Educational content focused on: i) the role of inflammation in asthma, ii) allergens that can trigger airway inflammation, iii) tips for avoiding or reducing exposure to triggers categorized as allergens (dust, bedding, furniture, pollens, food allergies, animal dander, mold, cockroaches), iv) caring for pillow and mattress covers, and v) good health practice (getting eight hours of sleep a day, drinking plenty of fluids, relaxing, eating a balanced diet and reducing stress).

#### Moderate intensity aerobic exercise

Those randomized to the moderate intensity aerobic exercise group completed a 12-week exercise training program at a frequency of 3 times per week, 30 minutes each session, at a steady-state intensity that achieved 60 – 75% of maximum heart rate (HR_max_). In order to determine each subject’s HR_max_ and fitness level, subjects performed a mandated graded treadmill test to volitional fatigue using a modified version of the Bruce protocol [14]; this test was performed at the UAB Clinical Exercise Facility. Subjects’ fitness levels were measured in the same manner at the conclusion of the 12-week intervention. Because subjects were using medications which may have influenced heart rate (such as bronchodilators), we utilized the graded treadmill test to allow us to measure subjects’ true maximum heart rates while taking their usual medications; Ratings of Perceived Exertion were recorded throughout the treadmill test. Maximal oxygen uptake in one minute (VO_2max_), as measured with a metabolic cart, was accepted as accurate if at least 2 of 3 physiologic criteria were met: leveling off of VO_2_ with increasing workload, respiratory exchange ratio (RER) > 1.15, and heart rate equal to age-predicted maximum. The target heart rate range was then calculated for each subject.

Recommended exercise prescription included a 5 minute warm-up, 30 minutes of steady state exercise via walking, and a 5 min cool-down; thus total exercise time was 40 minutes per exercise bout. Compliance with this prescription was verified via heart rate monitor readings as described below. The exercise program was performed at the UAB Recreational Center and was completed in conjunction with standard patient education described above.

### Subject visits

Subjects made three clinic visits to the UAB Lung Health Center. At the initial visit and prior to the start of the exercise protocol, all subjects underwent a complete physical with a board certified pulmonologist to ensure that the subjects were able to tolerate the exercise regimen. In addition, subjects completed health history and physical activity history questionnaires and documented asthma exacerbations at the initial visit. ECGs (12-lead) were used to permit safety monitoring of any previously un-diagnosed heart ailments and as part of the exercise testing for the subjects randomized to the exercise group. Lung function measurements and sample collection procedures (described below) were conducted at pre- and post-study intervals.

### Exercise monitoring

Subjects randomized to the exercise treatment group were monitored for adherence to the exercise prescription. Throughout the study, aerobic exercise subjects were asked to wear a Polar Heart Rate Monitor (model 625X), which stores relevant exercise history information, including heart rate target zones, exercise duration in target zones, average heart rate, maximum heart rate, and total exercise time. Staff instructed subjects in the use of the heart rate monitor at the initial visit. Stored information, including length of the exercise session and average target heart rate during the exercise session, was downloaded onto a computer at the post-study visits. In addition, subjects kept a weekly exercise diary, which included the frequency of exercise, asthma-related symptoms and exacerbations, the use of pillow and mattress covers, and good health practices. Subjects submitted the exercise logs to the study coordinator at the post-study visits. Sign-in logs from the participating fitness center were monitored weekly in order to verify physical activity logs of subjects randomized to the exercise group. Subjects were called each week to ensure they were recording the exercise activity and to encourage adherence. In addition, phone calls permitted investigator evaluation of any increases in asthma symptoms, other health problems that interfered with their exercise prescription, or problems with heart rate monitors. It also provided direct feedback about non-compliance.

It is possible that subjects in the exercise group may have exhibited improved asthmatic responses as a consequence of increased interaction with or attention from individuals at the fitness center. In order to control for this interaction / attention within the exercise group, individuals in the usual care group also received weekly phone calls from the study coordinator. During these brief phone calls, the study coordinator asked the subject how he/she was doing and if there was anything related to his/her respective program with which he/she needed assistance.

### Outcome indicators

The primary outcome indicator for this study was serum ECP. ECP is a marker of eosinophil activation found in both lavage fluids and serum of asthmatics. It has been demonstrated to correlate with asthma exacerbations and worsening as well as the effectiveness of asthma-related therapies [1,2]. Secondary outcome indicators included asthma control scores, airway and peripheral blood inflammatory markers (nasal lavage ECP, serum cytokines, peripheral blood immune cell populations), lung function parameters (FEV_1_, FEV_1_/FVC), and fitness measures (VO_2_ peak, HR peak, RER, total treadmill-time).

### Sample collection

Subjects provided blood and nasal lavage samples at the pre- and post-study visits. The post-study visit was conducted approximately 24 hours after the last session of exercise and at the same time of day in order to minimize effects of circadian rhythms on sample content [4].

#### Blood draw

Peripheral blood (15 ml at each visit) was collected in apyrogenic, heparinized tubes (Vacutainer, Becton Dickinson). Serum was separated and peripheral blood mononuclear cells (PBMCs) were isolated by density gradient centrifugation on Ficoll-Paque (Pharmacia). Serum samples were quick frozen in a dry ice bath and stored at −80°C until analysis; peripheral blood immune cells underwent immediate analyses.

#### Nasal lavage

Nasal lavage was performed with a disposable metered-dose pump filled with isotonic saline solution at room temperature [15]. Excessive mucus was first cleared by one spray of saline followed by a forceful exsufflation through the nostril. The same nostril was lavaged with 6 ml of the saline solution, which remained in the nasal cavity for approximately one minute and was then removed. Nasal lavage fluid (NLF) was then centrifuged to remove particulate matter and stored immediately at −80°C.

### Pro-inflammatory mediator analyses

Cytokine (ECP, IL-1β, IL-4, IL-5, IL-6, IL-13, TNFα) and total IgE content in serum and NLF were measured via enzyme linked immunosorbent assay (ELISA) according to the manufacturer’s instructions (BioSource).

### Cell differential analyses

Differential cell counts were performed on cells derived from peripheral blood as described previously [4]. Cell viability was determined via trypan blue exclusion and cell types were differentiated using Wright-Giemsa stain (Dade Behring Inc.). Cell differentials were determined from at least 500 leukocytes using standard hematological criteria.

### Asthma control

Subjects completed the Juniper Asthma Control Questionnaire (ACQ) at initial and post-intervention study visits. Asthma control was determined by the score on the ACQ [16]. This instrument integrates common indicators of asthma control, including use of bronchodilators, nocturnal symptoms, cough, activity level, and pulmonary function. It assesses the full range of clinical impairment that patients with asthma experience and is highly sensitive to small changes in asthma control that are clinically significant. Scores range from 0 to 6. Lower scores reflect better control, and a difference of greater than 0.5 between the pre-study score and the post-study score is considered clinically significant. Scores greater than or equal to 1.5 indicate poorly-controlled asthma with a positive predictive value of 0.88 [16].

### Pulmonary function analyses

Lung function was evaluated via spirometry using a portable Multispiro spirometer (Creative Biometrics) according to ATS/ERS guidelines [17]. Three forced vital capacity (FVC) maneuvers were performed for each subject and predicted values (FEV_1_, FVC) were determined.

### Statistical analysis

Baseline characteristics between sedentary and moderate intensity groups were compared. Given the small sample size of the pilot study, paired comparisons were made using Fisher’s exact test for nominal characteristics (gender, smoking, race) and Wilcoxon Rank Sum for continuous measures (age, FEV1, etc.). Given the longitudinal nature of the study and the repeated outcome measures per subject, repeated measures analysis of variance techniques were applied to examine changes over time and to determine if the changes in outcomes over time differed by groups. Because repeated measures analysis of variance assumes normally distributed outcomes, distributional properties of the residuals from the repeated measures analysis of variance models were examined. Across all outcomes, only minor deviations from the normality assumption were observed.

## Results

### Protocol design and subjects

Nineteen subjects were recruited into this pilot study; 16 completed the protocol (Figure 1; see Table 1 for baseline subject characteristics). At the start of the study, ninety-four percent of subjects had poor asthma control (ACQ) as indicated by the Asthma Control Questionnaire (ACQ score ≥ 1.5) [18]. None of the baseline characteristics, including gender, age, race, asthma control, asthma duration, and smoking history, differed significantly between the two treatment groups (Table 1).

**Table 1:** Baseline subject characteristics

	**Total**	**Moderate intensity (n = 6)**	**Education (n = 8)**	**p-value**
**Gender (% female)**	94	88	100	0.4
Mean Age (range)	53 (33–78)	53 (38–62)	54 (33–78)	1
Race (% non-white)	38	25	50	0.3
Mean BMI (range)	31.4 (17.2-48.4)	30.6 (23.9-39.2)	32.5 (17.2-48.4)	0.5
Mean Asthma Control (range)	2.3 (0.7-4)	1.9 (0.7-2.7)	2.6 (1.6-4)	0.3
Asthma duration ≥ 10 yrs (%)	32 (1–75)	25 (1–63)	40 (13–75)	0.2
Smoking History (%)	19	25	12	1

### Adherence to prescribed aerobic exercise protocols

Because of faulty heart rate monitor recordings, exercise data from two subjects were incomplete; therefore, these subjects were dropped from all study analyses. Completed data from heart rate monitors and exercise logs indicated that subjects in the exercise group, on average, performed 32 out of 36 of the prescribed exercise bouts. Of the completed exercise bouts, 80% of these bouts were performed for the prescribed duration and in the prescribed target heart rate range.

### Inflammatory mediators and circulating eosinophils

**Figure 2:**
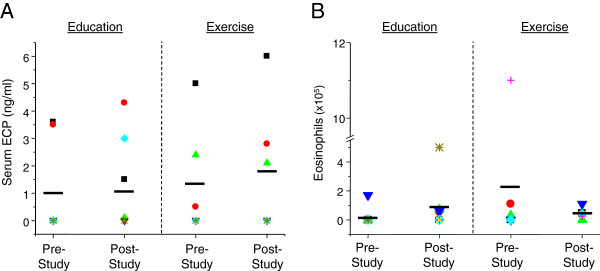
**Changes in circulating ECP and eosinophils between sedentary and exercise treatment groups.** Serum samples were collected from sedentary and exercise subjects at pre- and post-study timepoints. ECP levels in serum (A) were measured via ECP-specific ELISA. Cells were harvested from the peripheral blood at pre- and post-study timepoints. Differential cell counts for (B) eosinophils were performed as described in the text. Results are reported as percent of total peripheral blood immune cells. Black bars indicate average measurements in each group (education: n = 8; exercise: n = 6).

Figure 2 demonstrates that, at the post-study timepoint, subjects in both the sedentary and moderate intensity exercise groups exhibited no statistically significant differences in either circulating mediators, including serum ECP, or eosinophils. Subjects in the exercise group, however, exhibited a trend toward decreased eosinophilia, which was not observed in the sedentary subjects. No statistically significant differences were observed for the other circulating mediators, including IL-1β, TNFα, IL-4, IL-5, IL-6, and IL-13, as well as mediators in nasal lavage (data not shown).

### Asthma control

**Figure 3:**
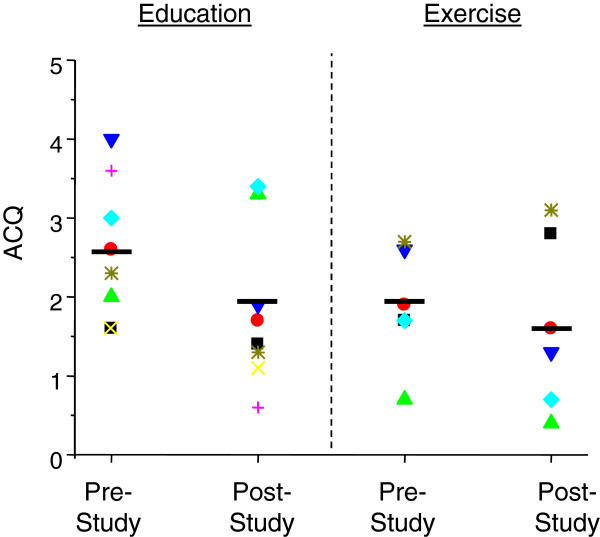
**Changes in asthma control between sedentary and exercise treatment groups.** Changes in asthma control were measured using the Asthma Control Questionnaire (ACQ). Black bars indicate average responses in each group (education: n = 8; exercise: n = 6).

Figure 3 indicates that subjects in the exercise group experienced a mean improvement in asthma control of 0.22 over the study period compared to a mean change of 0.73 in the sedentary control group. Although these changes were not statistically significant between groups, such changes did exhibit a trend toward improved asthma control within each group. The sedentary group displayed a pronounced placebo effect with a change greater than 0.5, which is considered clinically significant [16,18]. It should be noted that one patient in the exercise group did experience an exacerbation during her 12 weeks of exercise, but this exacerbation did not appear to be triggered by the exercise program. Her study data were eventually discarded secondary to faulty heart rate monitor recordings, thus the overall study data were not confounded by this exacerbation.

### Lung function parameters

**Figure 4:**
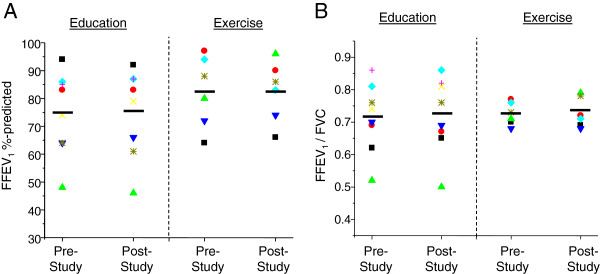
**Changes in lung function between sedentary and exercise treatment groups.** (A) FEV_1_ and (B) FEV_1_/FVC parameters were measured via spirometry in sedentary and exercise subjects at pre- and post-study timepoints. Results are reported as either percent predicted (FEV_1_) or percent actual (FEV_1_/FVC). Black bars indicate respective averages in each group (education: n = 8; exercise: n = 6).

Subjects in both treatment groups had post-bronchodilator spirometry performed at visits before and after completion of the study protocol; the FEV_1_ percent and the FEV_1_/FVC ratios of predicted for each subject were compared between these visits. Post-bronchodilator spirometry was chosen because home medications were not withheld prior to visits. As shown in Figure 4, there were no statistically significant changes in FEV_1_ percent and FEV_1_/FVC ratios for either treatment group.

### Fitness levels

**Figure 5:**
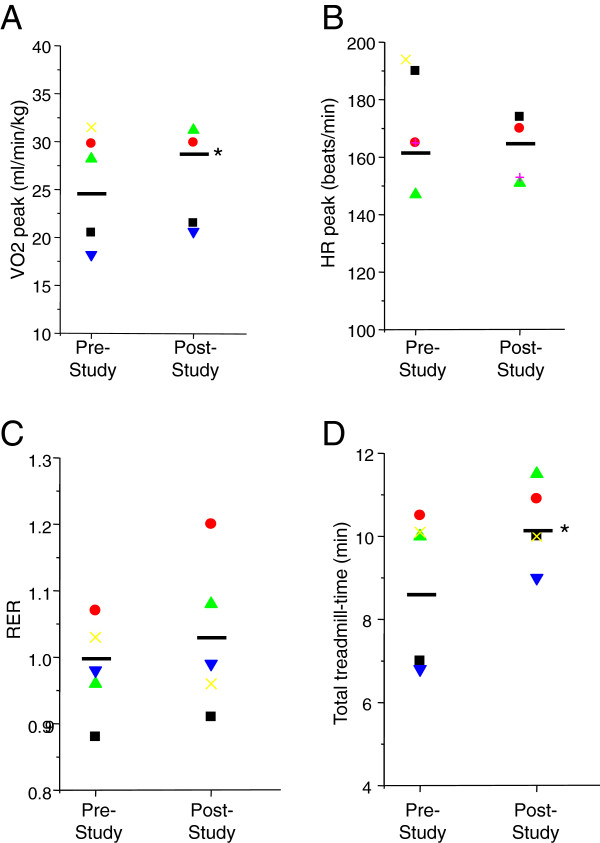
**Changes in fitness levels in exercise subjects.** Subjects randomized to the exercise treatment group performed a mandated graded treadmill test to volitional fatigue using a modified version of the Bruce protocol both before and upon protocol completion. Fitness measures included A) VO_2_ peak, B) HR peak, C) respiratory exchange ratio (RER), and D) total treadmill-time. Black bars indicate average responses in each group (*p ≤ 0.04 as compared with pre-study measurements; education: n = 8; exercise: n = 6).

Parameters chosen to measure changes in fitness levels for subjects in the exercise group pre- and post-protocol completion included VO_2_ peak, HR peak, respiratory exchange ratio (RER), and total treadmill time during exercise testing [19]. At protocol completion, subjects in the exercise group exhibited significant increases in VO_2_ peak (mean change 2.64) and total treadmill time (mean change 1.39 min) (Figure 5A, D); changes in RER (mean change 0.04) and HR peak (mean change) displayed a similar trend (Figure 5B, C). RER should be greater than or equal to 1.1 with intense exercise. None of our subjects, however, reached an RER of 1.1 on maximal exercise, possibly due to ventilation impairment.

## Discussion

The use of an exercise group and a sedentary group permitted direct comparison of the effectiveness of moderate intensity aerobic exercise plus education versus education only on asthma-related responses. Interventions which require a behavior change dictate the recruitment of motivated individuals; however, all subjects recruited to our study were informed that they could be randomized to the exercise group prior to signing consent. Although not all subjects were assigned to the exercise group, several potential biases (including differential attrition) which threaten the validity of a study design that includes a sedentary control group were addressed by recruiting only subjects who were willing to engage in a moderate intensity aerobic exercise protocol for twelve weeks.

Results suggest that exercise training at a moderate intensity improved asthma control and fitness measures in adult asthmatics; however, the final sample did not achieve sufficient statistical power to determine significant differences in most outcome measures. Because all subjects received education instruction in allergen avoidance, as well as pillow and mattress protectors, it is possible that the sedentary group may have included a greater proportion of atopic asthmatics; the atopic status of each of the subjects was not known. If this was indeed the case, the presence of atopy could have biased against the effect of exercise, since avoidance would have possibly improved asthma control and, thereby, diminished the observed effectiveness of the exercise intervention.

Although the majority of subjects self-reported having poorly controlled asthma, neither the mean circulating ECP levels nor eosinophil counts were elevated in these subjects. Despite this observation, subjects in the exercise group exhibited a trend toward decreased circulating eosinophils; however, the serum ECP levels in these subjects were unchanged. Such discordance between ECP levels and eosinophil counts may be due to exercise-mediated release of ECP from activated eosinophils as they traffic out of the circulation and into the vasculature, including the airway mucosa. Previous studies, which have reported elevation of serum ECP levels with a concomitant decrease in eosinophils following exercise, support this hypothesis. In these earlier studies, serum ECP levels and circulating eosinophils were measured during sessions of acute graded aerobic exercise [20] and endurance aerobic exercise at moderate altitude [21] in healthy subjects. In both studies, serum samples were collected within minutes post exercise. Both reports demonstrated that serum ECP levels were elevated while eosinophils counts decreased following the single, respective exercise session. In contrast, additional studies have demonstrated that physical activity has differential effects on the circulating levels of other cytokines, including IL-6 and TNFα [22-24]. These previous studies differ with the present study in exercise duration, frequency, subject fitness level, and/or timing of sample collection. As such, the differences in the observed effects of aerobic exercise on ECP and serum cytokine measurements between the present and previous studies are likely due to such exercise-related variables [25-30]; further, these differences underscore the need for additional study.

Completion and analyses of the current pilot study highlighted several areas that will need to be redefined in preparation for a future, larger study. ECP, which has been reported to correlate positively with asthma exacerbations and worsening [1,2], was chosen initially as a primary outcome measure because the objective of the future study is to test the functional consequences of aerobic exercise on asthmatic cellular and molecular responses. As observed in this current study, the choice of serum ECP as the primary outcome may have lacked sensitivity for assessing the effects of exercise on eosinophilic inflammation; therefore, sputum ECP levels and eosinophil counts, which better reflect the airway inflammation, will be used in the future study. In addition, revised inclusion criteria will require that subjects demonstrate eosinophilic inflammation at baseline in order ensure that any impact of exercise on this outcome can be observed. Baseline data will also include information on measures of subjects’ atopy in order to ensure that subjects with atopic responses are evenly distributed between the sedentary and exercise groups. Clinical outcome measures will be expanded to include additional lung function parameters, such as ventilatory capacity and exercise-induced bronchospasm (EIB). Previous results reported by Emtner and colleagues demonstrated that adults with mild-moderate asthma who underwent a high intensity exercise (80-90% predicted HR_max_) swimming program for 10 weeks exhibited increased ventilatory capacity, decreased EIB, and decreased asthma-related symptoms [31].

## Conclusions

Results from this pilot study suggest that aerobic exercise training at a moderate intensity may improve asthma control and fitness levels in the absence of asthma exacerbations in adult asthmatics. Strong adherence to the exercise protocol demonstrates the feasibility of the protocol in preparation for a larger, clinical trial that will test the effects of exercise on the cellular, molecular, and functional outcome measures of the asthmatic response. Such increased understanding will lead to the elucidation of the potential mechanisms that underlie the beneficial effects of moderate intensity exercise on asthmatic responses. Moreover, this understanding may lead to the development of novel therapeutic approaches, including the use of moderate intensity aerobic exercise as an adjunct therapy, for the treatment of this chronic disease.

## Abbreviations

HR_max_: Maximum heart rate; ECP: Eosinophilic cationic protein; ACQ: Asthma Control Questionnaire; FEV1: Largest volume of air expelled on forced expiration in 1 second; FVC: Forced vital capacity, largest volume of air that can be exhaled after maximal inspiration; FEV1/FVC: Ratio of the volume of air exhaled in 1 second to largest exhaled volume after maximal inspiration; AHR: Airway hyperresponsiveness; ACSM: American College of Sports Medicine; ATS: American Thoracic Society; UAB: University of Alabama at Birmingham; NAEPP: National Asthma Education and Prevention Program; COPD: Chronic obstructive pulmonary disease; VO_2max_: Maximal oxygen uptake in one minute; RER: Respiratory exchange ratio; ratio of the volume of exhaled carbon dioxide and inhaled oxygen in one breath; ECG: Electrocardiogram; HR peak: Highest recorded heart rate; PBMCs: Peripheral blood mononuclear cells; NLF: Nasal lavage fluid; TNF_α_: Tumor necrosis factor alpha; IgE: Immunoglobulin E.

## Competing interests

Dr. Mark Dransfield has received consulting fees from GlaxoSmithKline. None of the other authors have financial disclosures.

## Authors’ contributions

**AB** facilitated subject recruitment and monitoring, sample collection and analyses, data interpretation, and drafted the manuscript.

**CY** performed all of the statistical analyses.

**KE** processed and analyzed all samples collected throughout the study.

**SCT** facilitated subjects’ exercise testing and analyses of related results.

**LG** contributed to the design, implementation, and analyses of the study.

**MD** was responsible for the overall administration of the subject recruitment and study visits, conducted all of the subjects’ physicals for subjects, monitored adverse events, and worked with LMS to oversee study design and implementation.

**MB** contributed to the design, implementation, and analyses of the study.

**JB** contributed to the design, implementation, and analyses of the study.

**TPA** contributed to the design, implementation, and analyses of the study.

**LMS** initiated and oversaw the complete design, implementation, and data analyses of the study.

All authors read and approved the final manuscript.
